# Does consensus contour improve robustness and accuracy in ^18^F-FDG PET radiomic features?

**DOI:** 10.1186/s40658-024-00652-0

**Published:** 2024-06-06

**Authors:** Mingzan Zhuang, Xianru Li, Zhifen Qiu, Jitian Guan

**Affiliations:** 1 Department of Nuclear Medicine, Meizhou People’s Hospital, Meizhou, China; 2 Guangdong Engineering Technological Research Center of Clinical Molecular Diagnosis and Antibody Drugs, Meizhou People’s Hospital, Meizhou, China; 3 Department of Radiology, The Second Affiliated Hospital of Shantou University Medical College, Shantou, China

**Keywords:** Consensus contours, Radiomic analysis, Accuracy robustness, ^18^F-FDG PET imaging

## Abstract

**Purpose:**

The purpose of our study is to validate the robustness and accuracy of consensus contour in 2-deoxy-2-[^18^F]fluoro-D-glucose (^18^F-FDG) PET radiomic features.

**Methods:**

225 nasopharyngeal carcinoma (NPC) and 13 extended cardio-torso (XCAT) simulated data were enrolled. All segmentation were performed with four segmentation methods under two different initial masks, respectively. Consensus contour (ConSeg) was then developed using the majority vote rule. 107 radiomic features were extracted by Pyradiomics based on segmentation and the intraclass correlation coefficient (ICC) was calculated for each feature between masks or among segmentation, respectively. In XCAT ICC between segmentation and simulated ground truth were also calculated to access the accuracy.

**Results:**

ICC varied with the dataset, segmentation method, initial mask and feature type. ConSeg presented higher ICC for radiomic features in robustness tests and similar ICC in accuracy tests, compared with the average of four segmentation results. Higher ICC were also generally observed in irregular initial masks compared with rectangular masks in both robustness and accuracy tests. Furthermore, 19 features (17.76%) had ICC ≥ 0.75 in both robustness and accuracy tests for any of the segmentation methods or initial masks. The dataset was observed to have a large impact on the correlation relationships between radiomic features, but not the segmentation method or initial mask.

**Conclusions:**

The consensus contour combined with irregular initial mask could improve the robustness and accuracy in radiomic analysis to some extent. The correlation relationships between radiomic features and feature clusters largely depended on the dataset, but not segmentation method or initial mask.

**Supplementary Information:**

The online version contains supplementary material available at 10.1186/s40658-024-00652-0.

## Introduction

More recently, radiomic analysis in positron emission tomography (PET) imaging is of growing interest for quantitative assessment of tumor treatment response and prognosis [1, 2]. A typical challenge in PET imaging-based radiomic analysis is the lack of standard segmentation methods that could be robust and accurate against various imaging acquisition procedures in clinical scenarios [3, 4]. Although the variability and redundancy of radiomic features has been extensively explored, the results are varied and/or conflicting in many studies [5–10], which pose additional clinical challenges in radiomic analysis.

To overcome the inconsistency in tumor delineation and analysis, the consensus methods have been employed in many recent studies [11–15]. McGurk et al. [11] assessed consensus contour derived from different segmentation methods and found that consensus contour could improve accuracy and robustness compared with the varying performances of different segmentation methods. Schaefer et al. [12] also found that consensus contour could enhance robustness against the inconsistent performance of different segmentation results. Lv et al. [13] assessed the radiomics prognostic performance for patients with nasopharyngeal carcinoma (NPC) using the overlapping parts of two manual contours to derive radiomic features. Liang et al. [14] compared two radiomics tools for image analysis and clinical prediction with consensus agreement based on manual contour by three radiation oncologists.

In our previous companion study, we investigated the robustness and accuracy of consensus contour based on different individual segmentation results using clinical NPC cases and extended cardio-torso (XCAT) simulated tumors. Our results demonstrated that consensus contour could be a robust approach to mitigate segmentation variability, but did not appear to improve the segmentation accuracy on average [15]. Yet, on the basis of results published in the literature so far, it is still questioned to the impact of consensus contour from different PET segmentation methods on the robustness and accuracy for radiomic features. In this study, by focusing on the consensus contour, our aim is to provide a more clinically adaptable solution capable of achieving enhanced robust and accurate radiomic features in 2-deoxy-2-[^18^F]fluoro-D-glucose (^18^F-FDG) PET imaging.

## Methods

### XCAT simulation

**Fig. 1:**
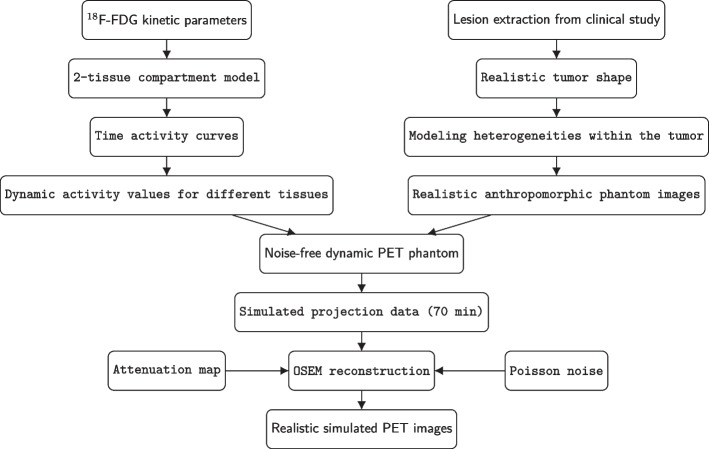
Flowchart illustrating various steps in the simulation of the realistic anthropomorphic model

The realistic anthropomorphic simulations were conducted using the XCAT phantom [16] and software for tomographic image reconstruction (STIR) [17] as built exactly the same as our previous study (Fig. 1) [15]. In this study the respiratory motion was not taken into consideration to avoid the influence of respiratory movement. The imaging matrix size for the simulated XCAT data was 200 $\times$ 200 with a voxel size of 0.50 $\times$ 0.41 $\times$ 0.41 cm^3^. In all, 13 tumors with heterogeneous uptake levels at the location of the right lung were simulated.

### Clinical NPC database

We reanalyzed PET/CT scans on 225 NPC patients who underwent ^18^F-FDG PET/CT scans at Meizhou people’s hospital from 2018 to 2020. Patient characteristics were previously described [15]. All PET images have a matrix size of 200 $\times$ 200 voxels with a voxel size of 0.30 $\times$ 0.41 $\times$ 0.41 cm^3^. The present study was approved by Meizhou people’s hospital ethics committee.

### Tumor segmentation

In PET images the boundaries of the primary tumors of NPC patients were delineated using four different segmentation methods: a method for automatic segmentation using an active contour model (MASAC) [18], an affinity propagation algorithm (AP) [19], the contrast-oriented thresholding method (ST) of Schaefer et al. [20], and segmentation using 41% of the maximum tumor value as a threshold (41MAX) [21]. The parameter of lambda in MASAC was set to 3 and the largest grouping was taken as the segmentation result for AP while keeping other parameters unchanged. Besides, all segmentation were also conducted with two different user-defined masks (rectangular and irregular cropping masks to roughly cover the tumor area, Supplemental Figure 1), repectively. Both two initial masks were manually defined by a clinical nuclear physician who also visually assessed whether the segmented contours were clinically acceptable and adjusted the initial masks as needed to exclude the high uptake area nearby. Finally, the consensus contour using the majority vote rule (Conseg) was derived from four different segmentation results [11, 12, 15].

### Extraction of radiomics features

The extraction of texture features was conducted using the Pyradiomics open-source python package (version 3.1) which was developed by van Griethuysen et al. [22]. It offers a reference standard for radiomic analysis and has been previously applied to several radiomics researches [23, 24]. For radiomics feature extraction, firstly, the original images were converted to standardized uptake value (SUV) images in which SUV was defined as the radioactivity concentration in a certain region normalized to the total injected dose and body weight of the patient. Then segmentation were performed using different approaches. Subsequently, a fixed binwidth of 0.25 units SUV was adopted for the calculations of radiomics features as taken by other studies [5, 9, 25]. No additional resampling or filters were applied in our study. Finally, 107 radiomic features were generated, including 14 shape-based, 18 first order statistics, 24 gray level co-occurrence matrix (GLCM), 16 gray level run length matrix (GLRLM), 16 gray level size zone matrix (GLSZM), 5 neighbouring gray tone difference matrix (NGTDM), and 14 gray level dependence matrix (GLDM) features (Supplemental Figures 2-15). The mathematical definitions and calculations of radiomic features could be found online (https://pyradiomics.readthedocs.io/en/latest/features.html). Most features were consistent with feature described by the Imaging Biomarker Standardization Initiative (IBSI) [8, 26–28], where feature definitions varying were specified as notes online by van Griethuysen et al. [22].

### Robustness and accuracy assessment

The intraclass correlation coefficient (ICC) was employed in our study, which has been widely used in robustness tests for quantitative studies [5, 9, 29]. Specifically, the ICC were computed for radiomic features between two different initial masks or among MASAC, AP, ST and 41MAX segmentation to compare the five segmentation methods, or two initial masks, respectively. In the simulated XCAT data, the ICC for radiomic features between segmentation and simulated ground truth (GT) were also calculated to access the accuracy of extracted radiomic features. For both robustness and accuracy tests, a two-way random effects model was adopted with single unit to measure the absolute agreement as the relationship between/among groups. The larger the value of ICC, the stronger the correlation between multiple variables. Some ICC were inadvertently estimated to be negative in this study due to the use of a small number of samples (13 cases) relative to the variability in the dataset [30, 31], and were neglected in our study because they were close to zero and less than 1% of the data (Supplemental Figures 2-15) [32, 33].

### Feature clustering

To minimize the redundancy of radiomic features, the Spearman’s correlation coefficients were usually adopted to assess the monotonic relationship between radiomic features for feature clustering [5], and then representative features were selected after clustering as candidates for classification or modelling. In this study, the Spearman’s correlation coefficients were employed as well to assess whether the relations between features and the composition of feature clusters were affected by the dataset, segmentation method, and/or initial mask.

### Data analysis

The calculation of ICC, Spearman’s correlation coefficients and their corresponding visualizations were performed with R 4.1.3 software [34]. Specifically, the ICC between multiple variables was classified with its absolute values as having repeatability that was excellent (≥ 0.9), good (0.75–0.89), moderate (0.5–0.74), or poor (<0.5) [35], and illustrated using a heatmap that displayed the ICC as a color-coded matrix with the color in the cell representing the strength and direction of the correlations. Besides, the triangle correlation heatmap was presented to show the Spearman’s correlation coefficients between each pairwise combination of radiomic features. The violin plot was employed as well, with its boundary representing the distribution of data and the centered dot at the middle symbolizing the median of the distribution. The median ICC of all features were also adopted as the criteria of robustness and accuracy in each subgroup in the results.

## Results

### Tumor contours

Representative contours by different segmentation methods under two initial masks for NPC and XCAT data were illustrated in Supplemental Figure 1. The median volume for five segmentation methods was 11.09 cm^3^ (range, 1.41–245.90 cm^3^) in NPC, and 16.64 cm^3^ (range, 3.45–56.40 cm^3^) in XCAT. The median maximum SUV for five segmentation methods was 12.45 with range 3.49–34.17 in NPC, and 6.87 with range 4.58–7.87 in XCAT.

### Robustness test

**Fig. 2:**
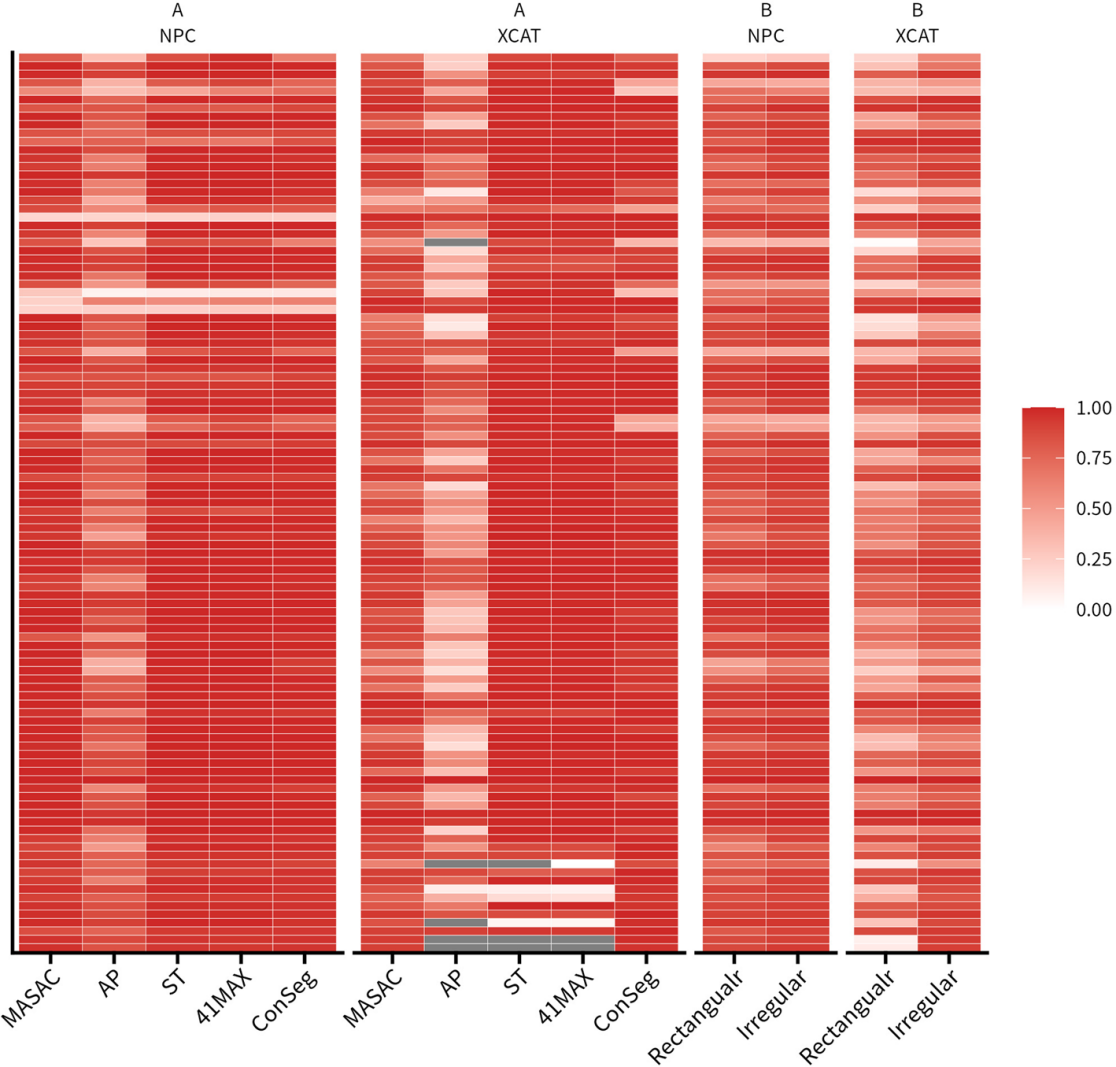
Heatmaps showing various intraclass correlation coefficients (ICC) for all radiomic features calculated based on two initial masks as function of segmentation method (**A**) and MASAC, AP, ST and 41MAX segmentation results as function of initial mask (**B**) in clinical NPC and XCAT simulation studies

**Fig. 3:**
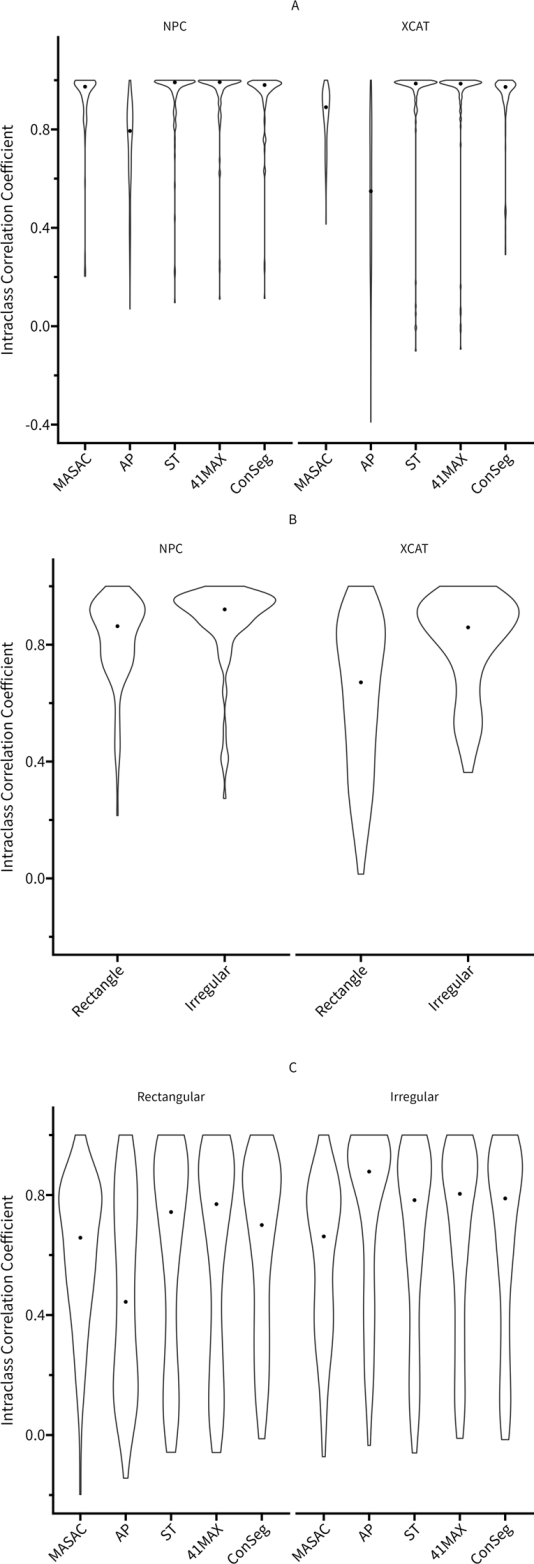
Violin plots showing various intraclass correlation coefficients (ICC) for all radiomic features calculated based on two initial masks as function of segmentation method (**A**), MASAC, AP, ST and 41MAX segmentation results as function of initial mask (**B**) as well as segmentation and ground truth as function of segmentation method and initial mask (**C**). The centered dot at the middle of the violin plot represents the median of the distribution

As illustrated in Figs. 2, 3 and Supplemental Figures 2-8, the ICC varied according to the dataset, segmentation method, initial mask and feature type. Overall, compared with XCAT the ICC of radiomics features showed higher average values in NPC for MASAC (9.37%), AP (44.62%), and both initial masks (Rectangular: 31.11%, Irregular: 8.36%), respectively. For both datasets in Figs. 2, 3 AP exhibited the lowest ICC (NPC: 0.79, XCAT: 0.55) across two different initial masks compared with other segmentation methods, whereas ConSeg showed similar high ICC values (NPC: 0.98, XCAT: 0.97) with ST and 41MAX. There were some differences however in MASAC that presenting poorer ICC (− 8.25%) in XCAT yet similar ICC in NPC compared with ConSeg.

As seen in Figs. 2, 3, Supplemental Figures 2-8 and Table 1 there were a greater proportion of ICC categorized as excellent (NPC: 85.05%, XCAT: 84.11%) in ConSeg compared with the average of four segmentation methods (NPC: 66.36%, XCAT: 57.94%) and AP (NPC: 16.82%, XCAT: 11.21%), respectively. Besides, ConSeg generally presented less poor parts in ICC (NPC: 2.80%, XCAT: 7.48%) compared with the average of four segmentation methods (NPC: 5.61%, XCAT: 13.08%), while AP had more poor parts (NPC: 13.08%, XCAT: 40.19%).

**Table 1:** Comparison of ICC calculated based on two inital masks for four segmentation methods, the average of four segmentation results and consensus contour

NPC	Excellent	Good	Moderate	Poor
MASAC	85	17	1	4
AP	18	49	26	14
ST	87	13	3	4
41MAX	92	9	3	3
Average	71	22	8	6
ConSeg	91	9	4	3

XCAT	Excellent	Good	Moderate	Poor
MASAC	49	38	19	1
AP	12	24	28	43
ST	92	9	0	6
41MAX	94	6	1	6
Average	62	19	12	14
ConSeg	90	8	1	8

Furthermore, it could be summarized from Figs. 2, 3, Supplemental Figures 2-8 and Table 2 that irregular initial masks produced more excellent ICC (NPC: 60.75%, XCAT: 36.45%) compared to rectangular masks (NPC: 39.25%, XCAT:14.95%). Irregular masks showed similar amount of poor ICC in NPC but much better results in XCAT (7.47%) compared with rectangular masks (30.56%).

In addition, 19 features (17.76%) were found to be independent of initial mask and segmentation method (ICC ≥ 0.75) for both datasets (Table 3). Among them, the ICC of X90Percentile, Energy, Maximum, and TotalEnergy in first order statistics were found to ≥ 0.9 against different initial masks or segmentation methods for both datasets (Table 3 and Supplemental Figure 3).

**Table 2:** Comparison of ICC calculated based on MASAC, AP, ST and 41MAX for two initial masks

NPC	Excellent	Good	Moderate	Poor
Rectangular	42	39	20	6
Irregular	65	30	6	6

XCAT	Excellent	Good	Moderate	Poor
Rectangular	16	29	29	33
Irregular	39	36	24	8

### Accuracy test

**Fig. 4:**
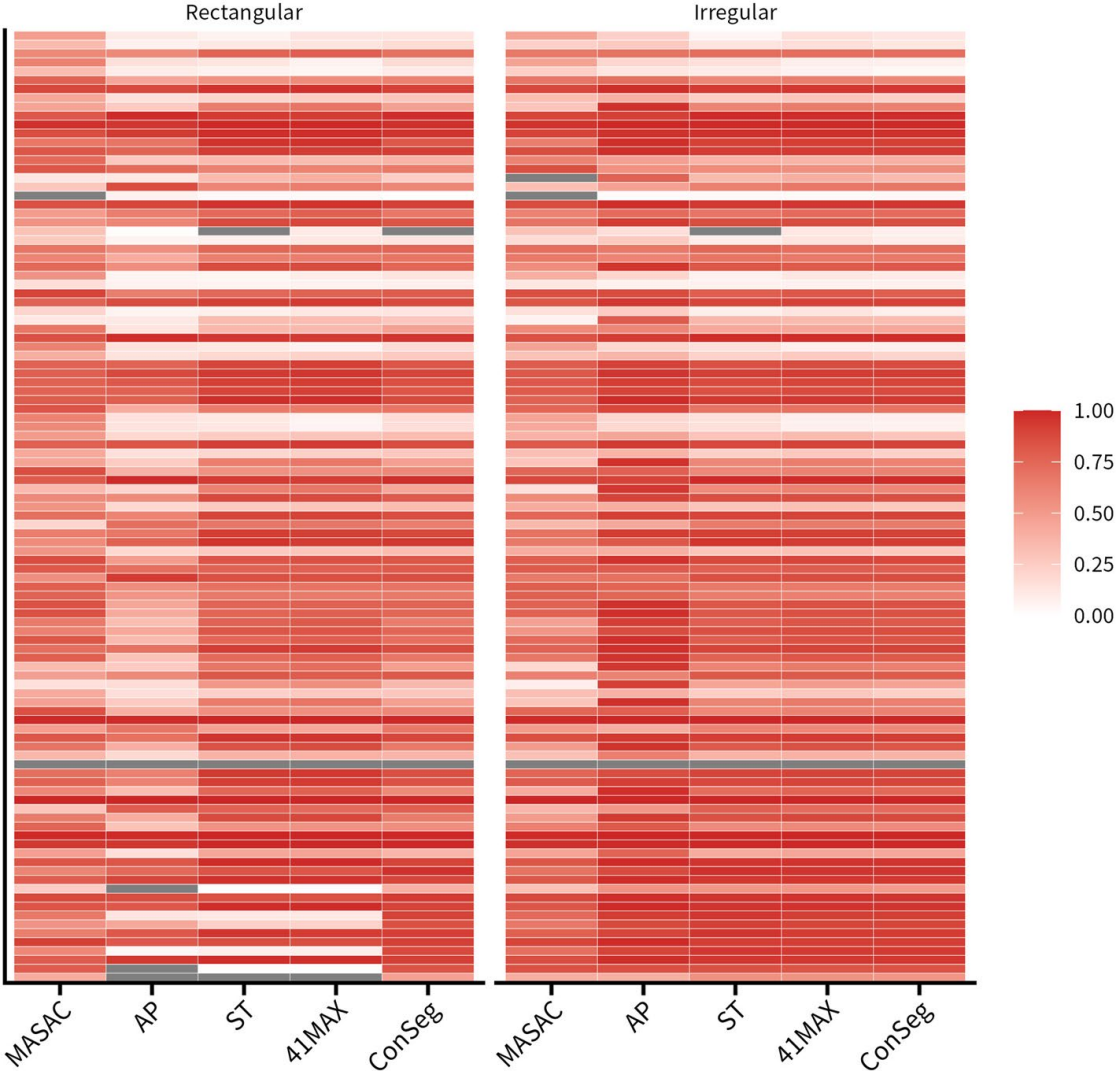
Heatmaps showing various intraclass correlation coefficients (ICC) for all radiomic features calculated based on segmentation and ground truth as function of segmentation method and initial mask in XCAT simulation studies

As for the ICC calculated based on segmentation and GT, ConSeg was found to be similar with 41MAX and ST regardless of initial masks in Figs. 3, 4 and Supplemental Figures 9-15. Although AP presented higher ICC (0.89) in irregular masks compared with other segmentation methods (MASAC: 0.66, ST: 0.78, 41MAX: 0.80, ConSeg: 0.79), the lowest ICC was also seen for AP (0.44) with rectangular masks among different segmentation methods (MASAC: 0.66, ST: 0.74, 41MAX: 0.77, ConSeg: 0.70).

**Table 3:** 19 selected radiomic features showing ICC ≥ 0.75 for all segmentation methods and initial masks in both robustness and accuracy tests

Class	Feature
Shape	LeastAxisLength
	Maximum2DDiameterColumn
	MinorAxisLength
	SurfaceArea
First Order	X90Percentile ^a^
	Energy ^a^
	Maximum ^a^
	TotalEnergy ^a^
GLCM	Imc1
	Imc2
GLRLM	GrayLevelNonUniformity
	LongRunEmphasis
	RunLengthNonUniformityNormalized
	RunPercentage
	RunVariance
	ShortRunEmphasis
GLSZM	GrayLevelNonUniformity
GLDM	DependenceNonUniformityNormalized
	LargeDependenceEmphasis

^a^ ICC ≥ 0.90 for all segmentation methods and initial masks in both robustness and accuracy tests

**Fig. 5:**
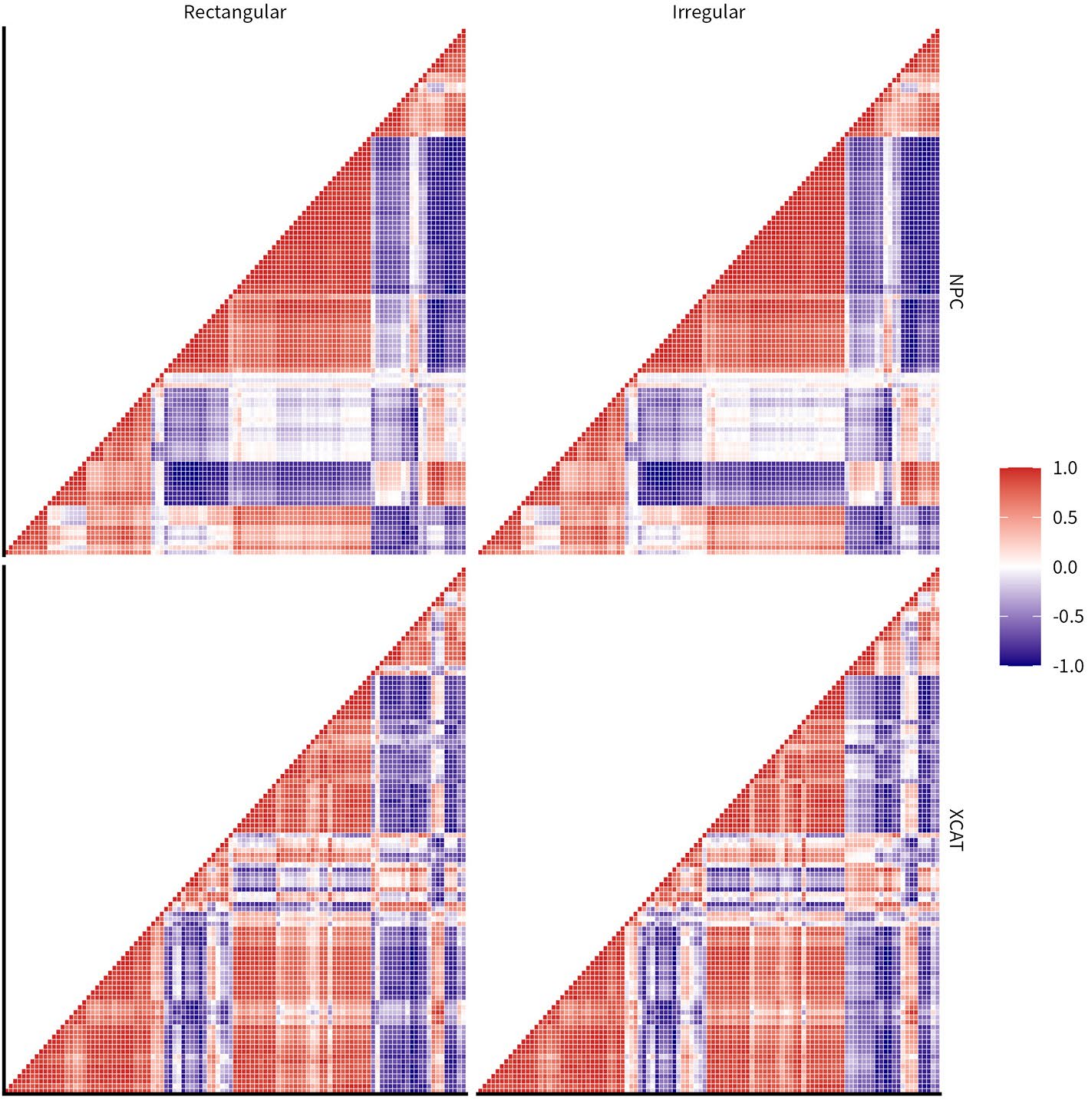
Heatmaps showing Spearman’s correlation coefficients calculated between radiomic features as function of dataset and initial mask with ConSeg segmentation. The feature order in correlation matrics was set according to the correlation coefficients for ConSeg with irregular mask in NPC dataset

Besides, it could be found in Table 4 that ConSeg showed similar ICC across two initial masks compare with the average of four segmentation methods, and the ICC for radiomics features generally presented better results for ConSeg in irregular masks (Excellent: 28.97%, Poor: 26.17%) than rectangular masks (Excellent: 19.63%, Poor: 32.71%). Similar results could also be found in the average of four segmentation methods for irregular masks (Excellent: 28.04%, Poor: 28.97%) compared with rectangular masks (Excellent: 18.69%, Poor: 36.44%).

**Table 4:** Comparison of ICC calculated based on segmentation and ground truth for four segmentation methods, the average of four segmentation results and consensus contour

Rectangular	Excellent	Good	Moderate	Poor
MASAC	6	37	32	32
AP	11	19	22	55
ST	29	23	21	34
41MAX	32	23	18	34
Average	20	26	23	39
ConSeg	21	31	20	35

Irregular	Excellent	Good	Moderate	Poor
MASAC	5	38	24	40
AP	51	17	13	26
ST	29	27	23	28
41MAX	33	23	23	28
Average	30	26	21	31
ConSeg	31	24	24	28

In general, radiomic features that were more stable in robustness tests were also found to be more accurate in accuracy tests. Specifically, the same 19 features (17.76%) were found to be robust (ICC ≥ 0.75) against either segmentation method or initial mask, and the ICC in X90Percentile, Energy, Maximum and TotalEnergy in first order statistics, and DependenceVariance in GLDM were shown to be ≥ 0.90 in the accuracy test for any of the segmentation methods or initial masks (Table 3 and Supplemental Figures 10, 15).

### Interaction between radiomic features

The Spearman’s correlation coefficients matrixes between radiomic features for ConSeg segmentation were shown in Fig. 5, and the corresponding matrixes for other segmentation methods were appended as supplementary data (Supplemental Figures 16-19). In order to present the differeces in correlation, the feature order in correlation matrixes for ConSeg with irregular mask in NPC (in the uper left corner of Fig. 5) was adopted to illustrate the correlation matrixes of other settings. It could be found that different segmentation methods and initial masks had little influence on the correlation matrix. Despite that, the changing of datasets posed a much larger impact on the correlation matrixes between radiomic features and the corresponding feature clusters as a consequence.

## Discussion

A confounding issue in radiomic analysis for medical imaging is the variation of results for the repeatability and dimensionality reduction in radiomic features, and the variation creates difficulties in the standardization and verification, making it difficult to develop a meaningful and clinically adaptable solution for radiomic analysis [5–10, 36]. Kocak et al. [37] also investigated the reliability of radiomic features based on consensus segmentation in MR and CT images, and consensus segmentation was found to have significant reliability issues in radiomic analysis, suggesting careful reliability validation on consensus segmentation should be performed before clinical use.

To our knowledge, our study provided the first quantitative assessment on the robustness and accuracy of consensus contour in radiomic analysis using PET imaging. We investigated potential factors that affected radiomic features in this study and focused on establishing an optimal solution for reproducible radiomic analysis in clinic. It was found that although the ICC varied with the dataset, segmentation method, initial mask and feature type, consensus contour combined with irregular initial mask could improve the robustness and accuracy in radiomic analysis to some extent, which could be eventually applicable in clinical settings without increasing workload due to the possibility to extend multiple auto segmentation methods in workstations. The results in this study were also consistent with our previous study demonstrating that consensus contour could enhance the robustness in PET imaging segmentation [15]. Additionally, the correlation relationships between radiomic features as well as feature clusters were found to be highly influenced by the dataset, but not segmentation method or initial mask.

Based on these findings, we believe that the highly varied or even conflicting results in radiomic analysis could be explained by the use of different datasets, segmentation procedures and/or investigated feature types. Similar findings were also observed in some studies examining the robustness of radiomic features [3, 5, 10, 38]. Traverso et al. [38] evaluated 41 studies on the repeatability and reproducibility of radiomic features and found that the repeatability of radiomic features are varied at various degrees with image acquisition settings, reconstruction protocol, image preprocessing, and software to derive radiomic features. Pfaehler et al. [5] explored the repeatability of radiomic features in PET images with different reconstruction protocols, noise and segmentation methods, and found that feature repeatability and space reduction depended on all investigated factors, suggesting the standardization of image acquisition and preprocessing before clinical application. Eertink et al. [10] assessed the discriminative power of radiomics features with different segmentation methods, and showed that no substantial difference among the segmentation methods, in which the observations were consistent with our results to some extent that showing the segmentation method had minor impact on the relationships between radiomic features.

In our study a majority of the investigated features showed varied ICC for different segmentation methods or initial masks, which recapitulated the need to have careful feature selection in radiomic analysis. More specifically, 19 features (17.76%) were found to be robust against different segmentation methods or initial masks in either robustness or accuracy tests. It should be noted that those features, such as contrast, coarseness, busyness, homogeneity, entropy, dissimilarity (mathematically equal to DifferenceAverage in this study), high-intensity emphasis (names as HighGrayLevelEmphasis in this study), and zone percentage, that have been previously considered as reliable candidates in tumor discrimination [3, 39, 40], presented large variations in our results (ICC < 0.75 in at least one of the robustness and/or accuracy tests). Similar results could also be observed in other studies [5, 41]. Therefore, the features employed to quantify changes in response to therapy should be carefully selected and reviewed.

Of the 19 selected radiomic features, X90Percentile, Energy, Maximum and TotalEnergy in first order statistics were found to have excellent reliability against different segmentation methods or initial masks in both robustness and accuracy tests, indicating these features might be better candidates for reproducible radiomic analysis. The first order features were also reported to be more reproducible than shape and textural features in general [38, 41]. Despite that, it is worth noting that TotalEnergy is the value of Energy feature scaled by the volume of the voxel, highly corelating with Energy by definition, and X90Percentile is the 90th percentile of the voxels within segmentation that is also closely linked to Maximum. Therefore, it seems more reasonable to adopt one or two of them for radiomic analysis in clinic, such as Energy and/or Maximum.

As a point of caution, it must be emphasized that the radiomic feature values we observed in PET images differ from those in reality (real tumor) due to the processes of image projection, reconstruction and the corresponding noise. In our previous study [40], we found that most assessed radiomic features were significantly different in either SUV or Ki images compared with those obtained from the noise-free ground truth. Pfaehler et al. [9] also proposed that careful consideration should be given to small lesions which may not reflect their actual heterogeneity information in PET images.

One of the limitations of this study was that there was no statistical comparisons between groups in our radiomic analysis. This is because the relationships between radiomic features may vary under different scenarios as shown in our results and so only heatmaps and violin plots were adopted to display the results to avoid the bias in the analysis. Moreover, we did not study the influence of respiratory motion on consensus contour in radiomic analysis. Adachi et al. [42] evaluated the influence of respiratory motion on the robustness of radiomic features for four-dimensional CT images using an anthropomorphic chest phantom and found that the amplitude of target motion <1 mm could help to the robustness of radiomic features. Xu et al. [43] investigated the impact of respiratory motion on radiomic features in ^18^F-FDG PET imaging and showed that respiratory motion had considerable impact on feature stability and optimizing preprocessing configuration may help to improve feature stability and diagnostic performance. Besides, the absence of ground truth in NPC dataset does not allow the assessment of data accuracy, and the simulated cases may also not be sufficient to fully illustrate the data accuracy in radiomic analysis. Our study is also limited to a single center with relatively homogeneous populations. Therefore, further studies with more heterogeneous data would be needed to assess whether the conclusion is valid under different scenarios. Additionally, slightly different interpolated results for some radiomic features may be caused due to differences between Pyradiomics and IBSI regarding pre-processing [26, 44]. These differences are likely to be small and documented online (https://pyradiomics.readthedocs.io/en/latest/faq.html).

## Conclusions

The present study demonstrated that although the ICC for radiomic features were sensitive to the dataset, segmentation method, initial mask and feature type, the consensus contour combined with irregular initial mask could improve the robustness and accuracy in radiomic analysis to some extent, which might be the optimal solution for reproducible radiomic analysis in clinic. In addition, 19 features with low level of variations under different segmentation methods and initial masks in either robustness or accuracy tests were identified as well, and the correlation relationships between radiomic features as well as feature clusters were found to be highly influenced by the dataset, but not segmentation method or initial mask.

### Supplementary Information

Supplementary file1

Supplementary file2

## Data Availability

The datasets used and analysed during the current study are available from the corresponding author on reasonable request.
