# Supplementary material for: Does consensus contour improve robustness and accuracy in 18F-FDG PET radiomic features?
Source: EJNMMI Phys. 2024 Jun 6;11:48. doi: 10.1186/s40658-024-00652-0 (PMC11153434; doi:10.1186/s40658-024-00652-0)
Supplement: Supplementary file 1 — Supplementary file1 [file 40658_2024_652_MOESM1_ESM.pdf]

Additional Files

Supplemental Figure

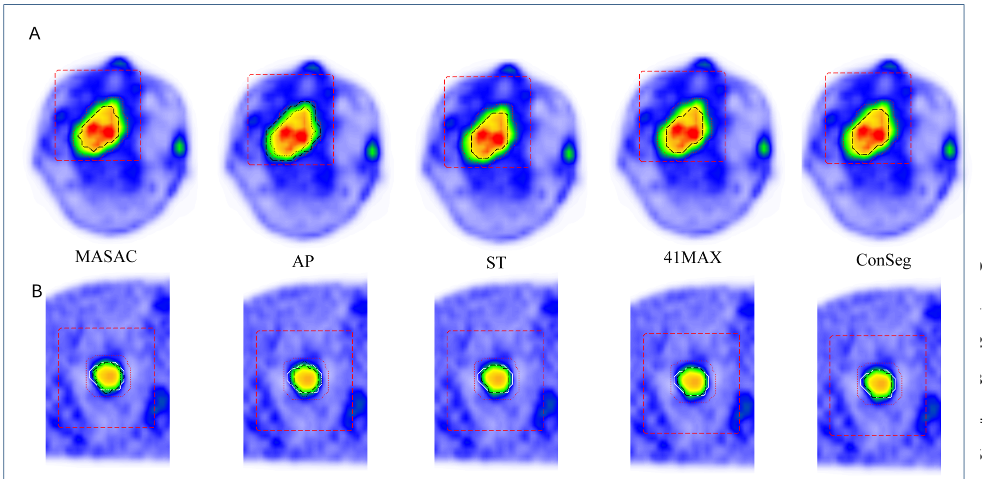

Figure 1 Representative contours of five different methods (black dashed horizontal line: segmentation with rectangular mask, black dotted line: segmentation with irregular cropping mask, white: simulated ground truth) with two initial masks (red dashed horizontal line: rectangular mask, red dotted line: irregular cropping mask) in clinical NPC (A) and XCAT (B) simulation studies.

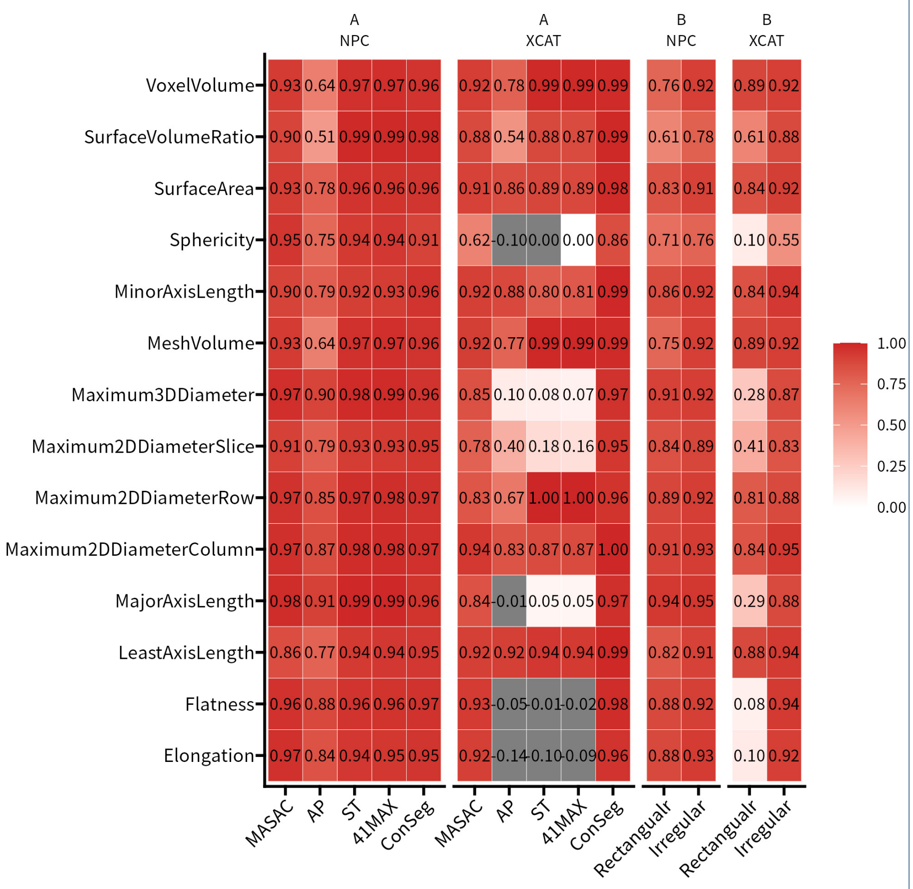

Figure 2 Heatmaps showing various intraclass correlation coefficients (ICC) for shape features calculated based on two initial masks as function of segmentation method (A) and MASAC, AP, ST and 41MAX segmentation results as function of initial mask (B) in clinical NPC and XCAT simulation studies.

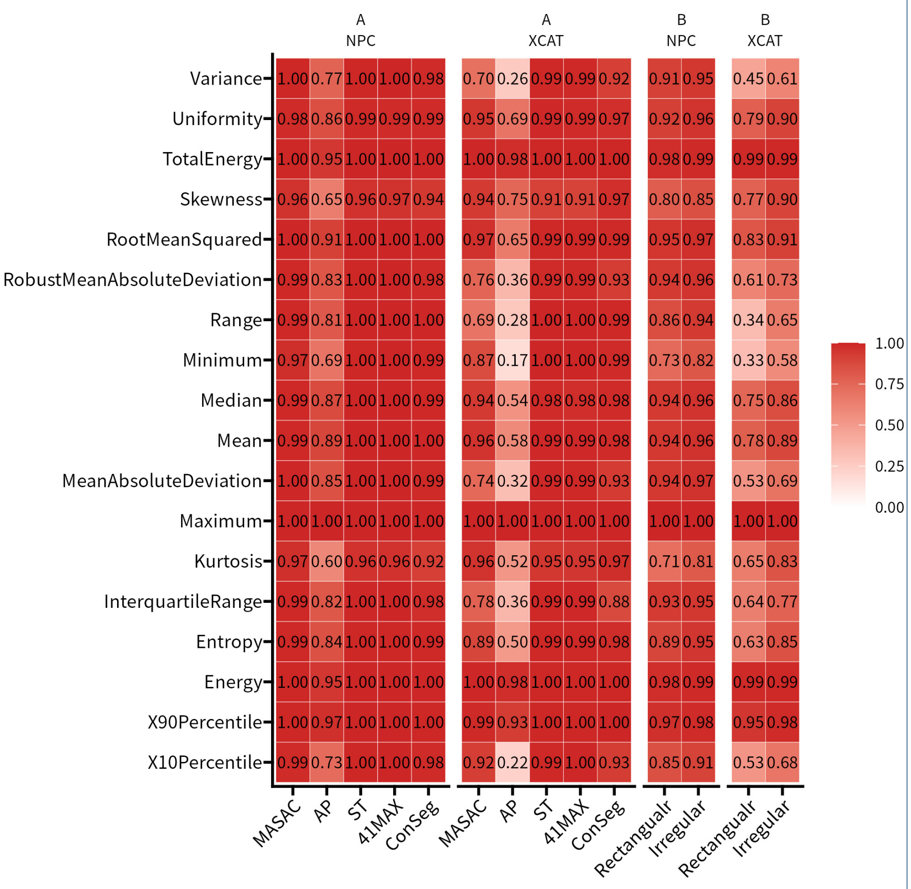

Figure 3 Heatmaps showing various intraclass correlation coefficients (ICC) for first order features calculated based on two initial masks as function of segmentation method (A) and MASAC, AP, ST and 41MAX segmentation results as function of initial mask (B) in clinical NPC and XCAT simulation studies.

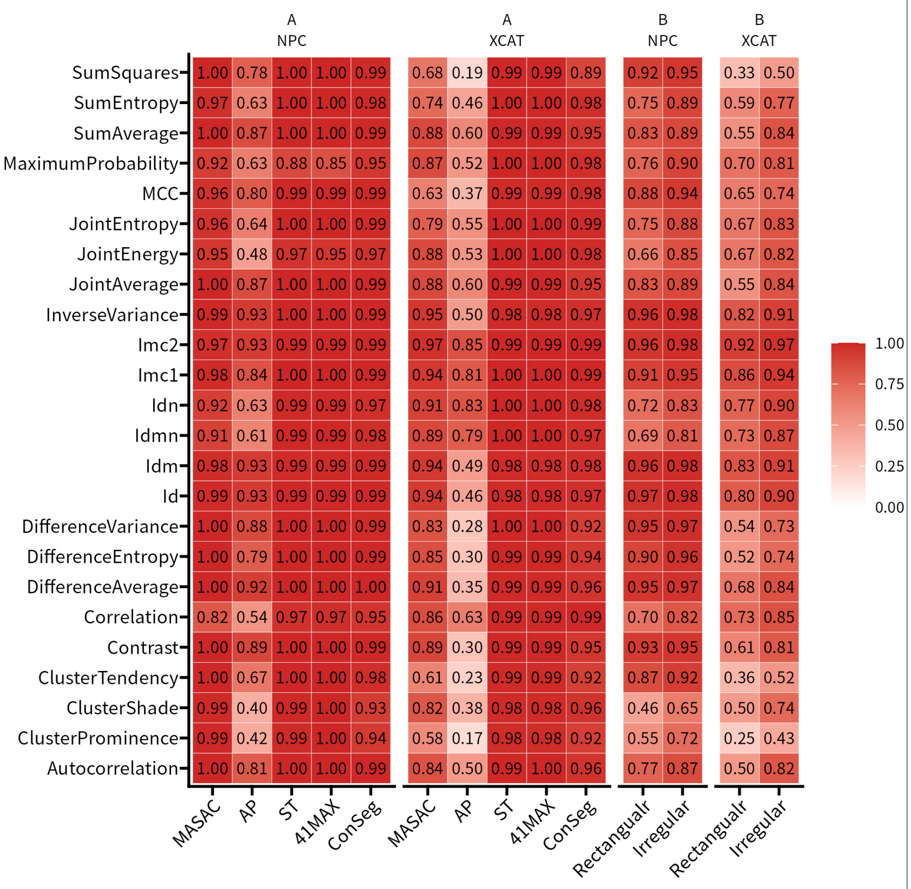

Figure 4 Heatmaps showing various intraclass correlation coefficients (ICC) for GLCM features calculated based on two initial masks as function of segmentation method (A) and MASAC, AP, ST and 41MAX segmentation results as function of initial mask (B) in clinical NPC and XCAT simulation studies.

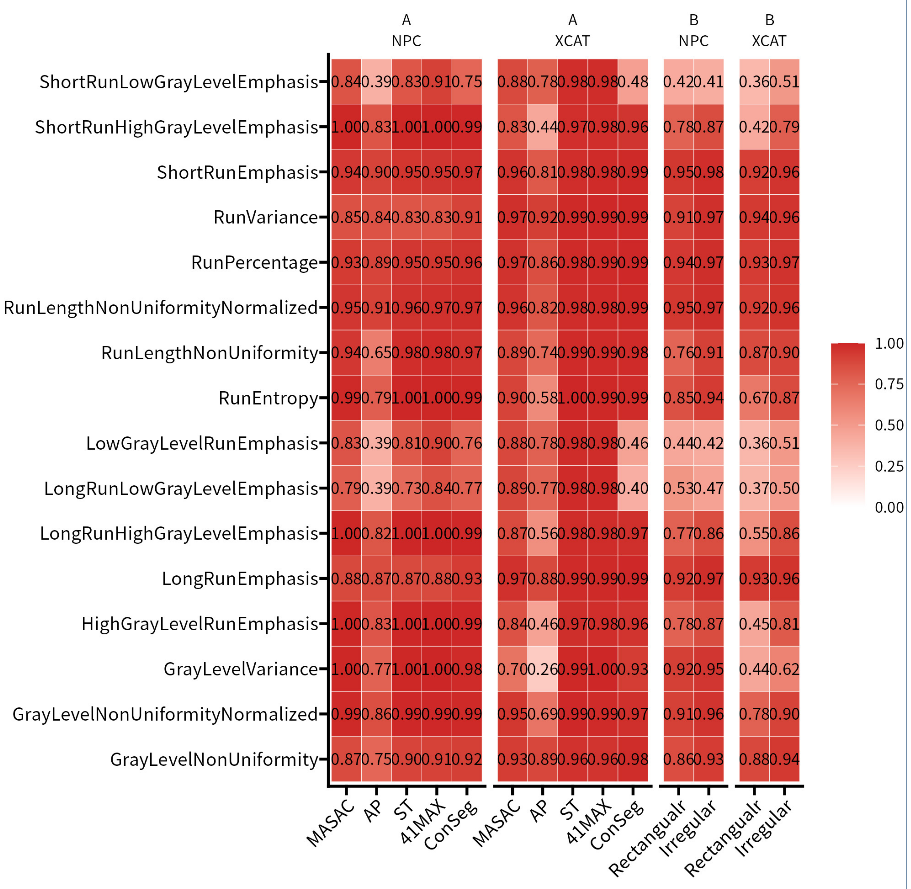

Figure 5 Heatmaps showing various intraclass correlation coefficients (ICC) for GLRLM features calculated based on two initial masks as function of segmentation method (A) and MASAC, AP, ST and 41MAX segmentation results as function of initial mask (B) in clinical NPC and XCAT simulation studies.

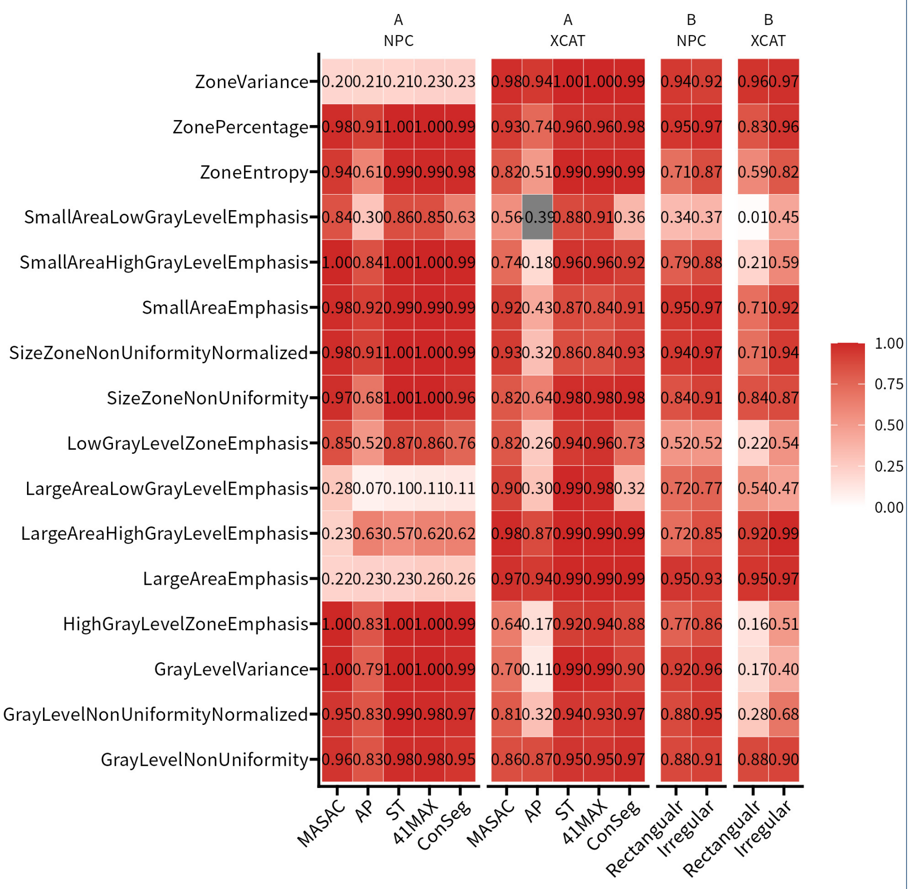

Figure 6 Heatmaps showing various intraclass correlation coefficients (ICC) for GLSZM features calculated based on two initial masks as function of segmentation method (A) and MASAC, AP, ST and 41MAX segmentation results as function of initial mask (B) in clinical NPC and XCAT simulation studies.

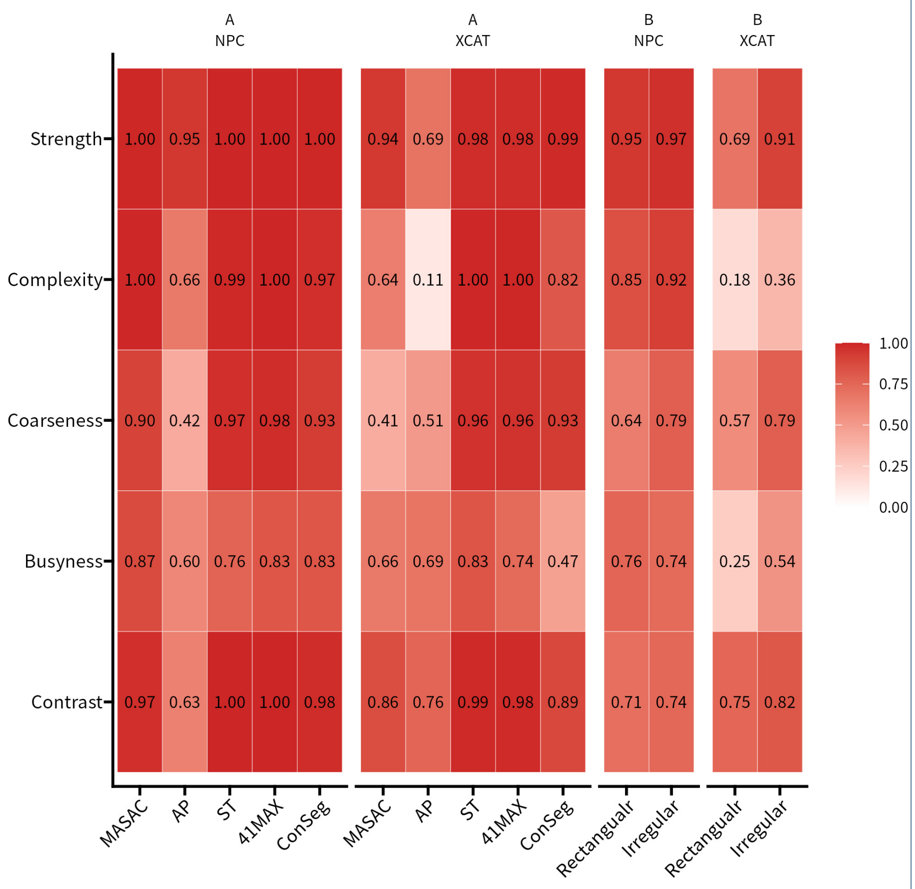

Figure 7 Heatmaps showing various intraclass correlation coefficients (ICC) for NGTDM features calculated based on two initial masks as function of segmentation method (A) and MASAC, AP, ST and 41MAX segmentation results as function of initial mask (B) in clinical NPC and XCAT simulation studies.

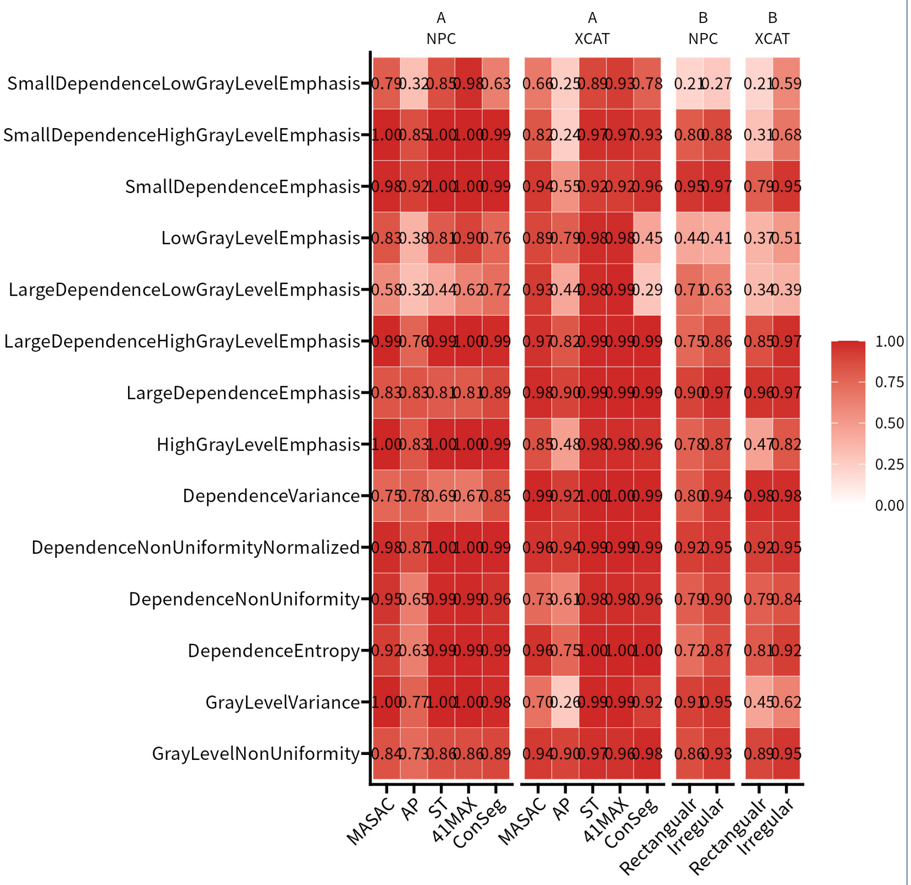

Figure 8 Heatmaps showing various intraclass correlation coefficients (ICC) for GLDM features calculated based on two initial masks as function of segmentation method (A) and MASAC, AP, ST and 41MAX segmentation results as function of initial mask (B) in clinical NPC and XCAT simulation studies.

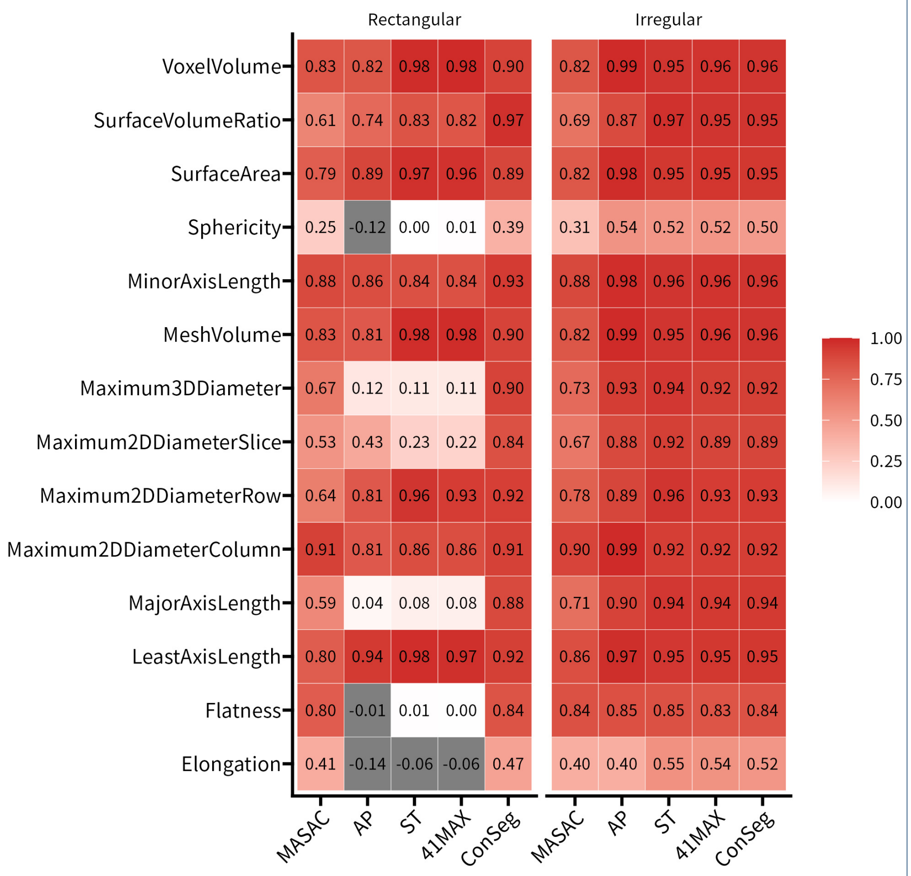

Figure 9 Heatmaps showing various intraclass correlation coefficients (ICC) for shape features calculated based on segmentation and ground truth as function of segmentation method and initial mask in XCAT simulation studies.

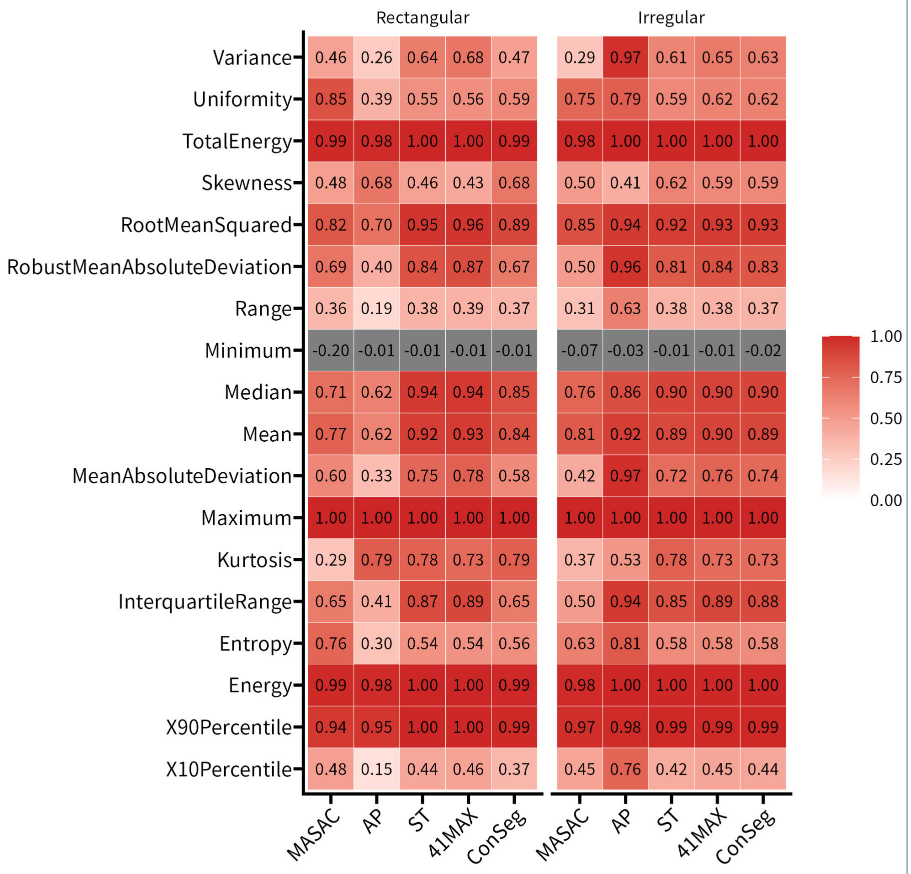

Figure 10 Heatmaps showing various intraclass correlation coefficients (ICC) for first order features calculated based on segmentation and ground truth as function of segmentation method and initial mask in XCAT simulation studies.

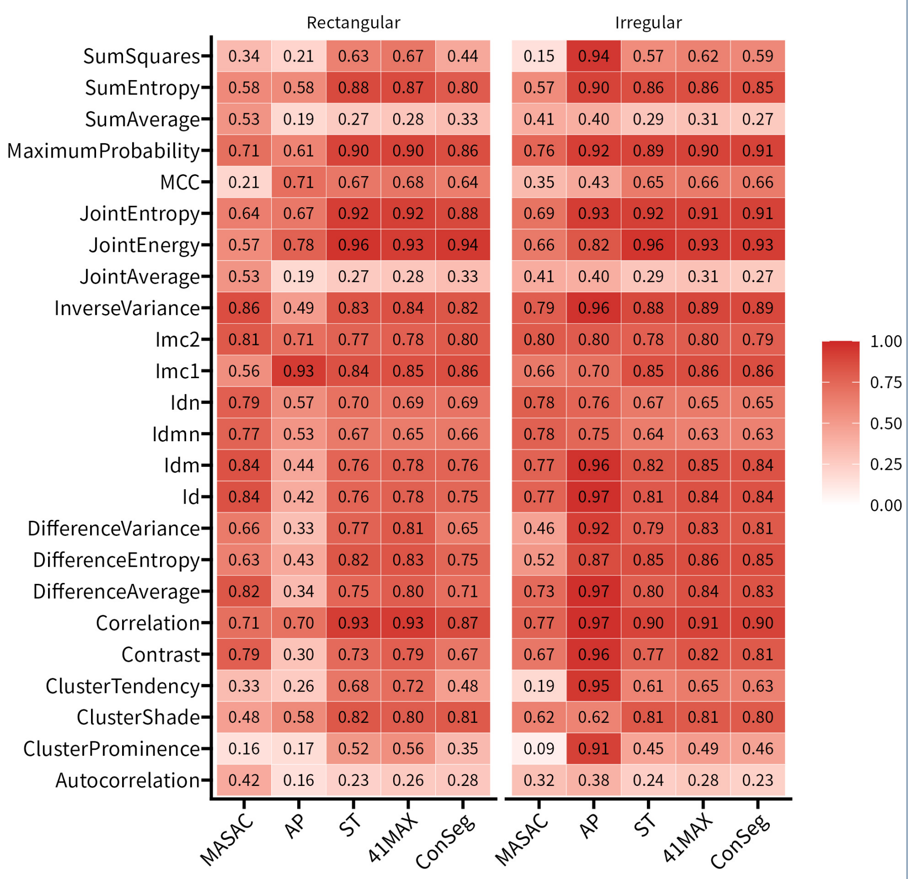

Figure 11 Heatmaps showing various intraclass correlation coefficients (ICC) for GLCM features calculated based on segmentation and ground truth as function of segmentation method and initial mask in XCAT simulation studies.

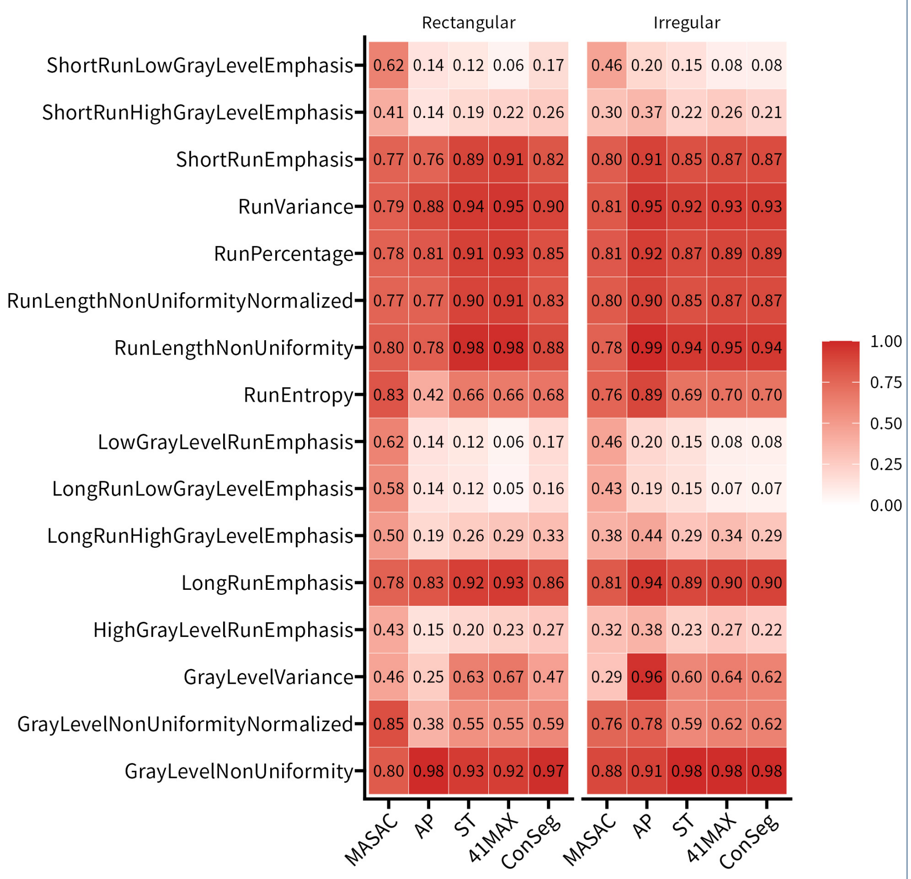

Figure 12 Heatmaps showing various intraclass correlation coefficients (ICC) for GLRLM features calculated based on segmentation and ground truth as function of segmentation method and initial mask in XCAT simulation studies.

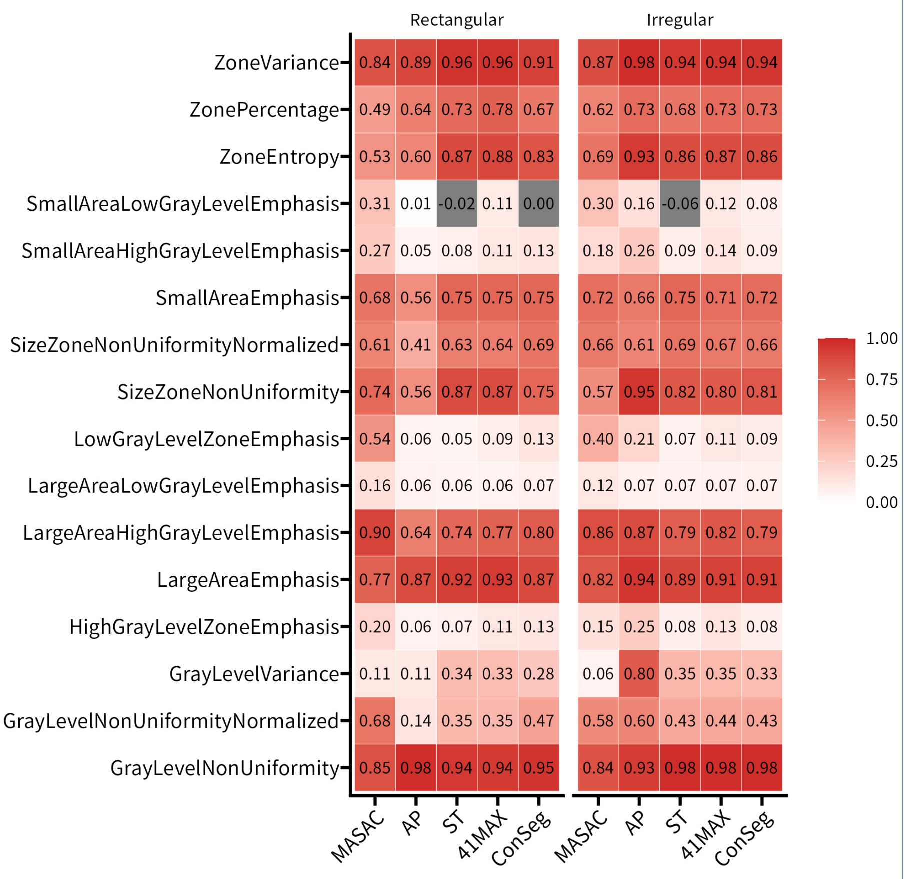

Figure 13 Heatmaps showing various intraclass correlation coefficients (ICC) for GLSZM features calculated based on segmentation and ground truth as function of segmentation method and initial mask in XCAT simulation studies.

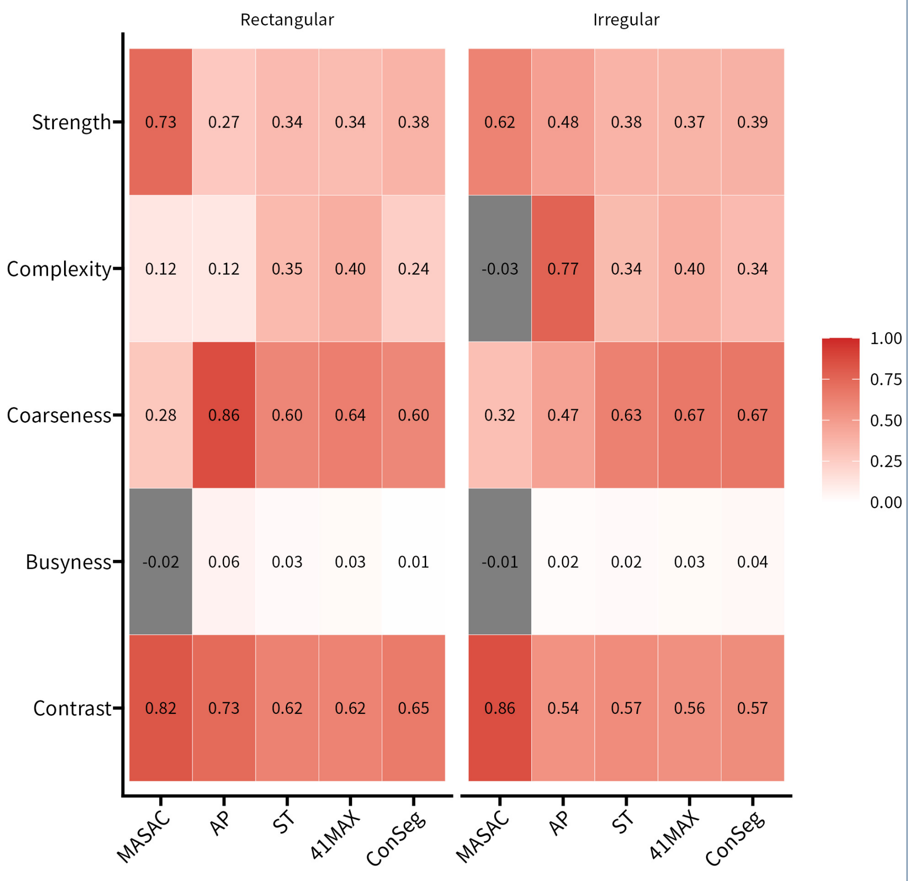

Figure 14 Heatmaps showing various intraclass correlation coefficients (ICC) for NGTDM features calculated based on segmentation and ground truth as function of segmentation method and initial mask in XCAT simulation studies.

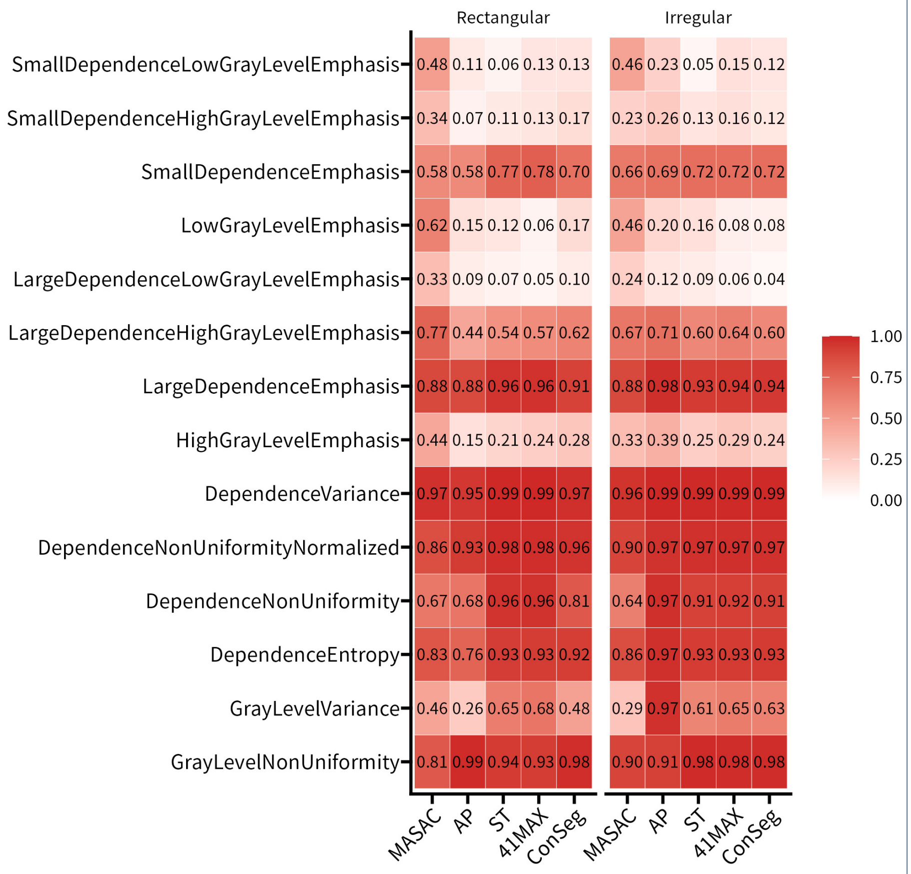

Figure 15 Heatmaps showing various intraclass correlation coefficients (ICC) for GLDM features calculated based on segmentation and ground truth as function of segmentation method and initial mask in XCAT simulation studies.

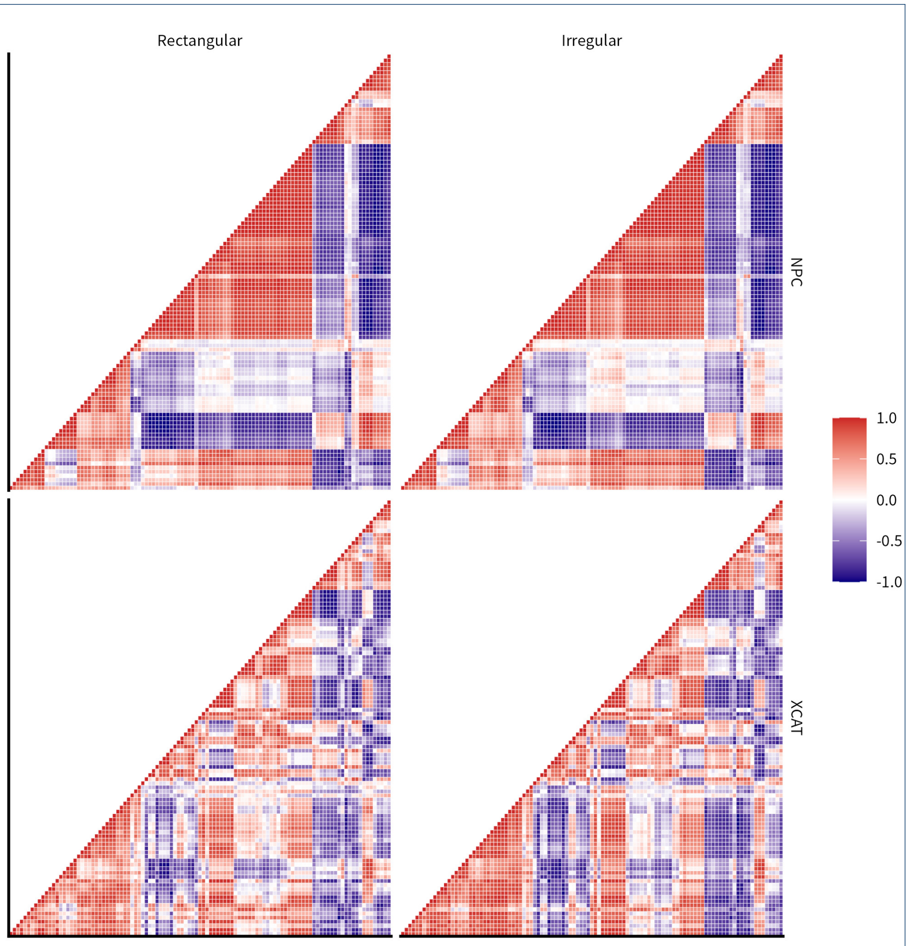

Figure 16 Heatmaps showing Spearman's correlation coefficients calculated between radiomic features as function of dataset and initial mask with MASAC segmentation. The feature order in correlation matrics was set according to the correlation coefficients for ConSeg with irregular mask in NPC dataset.

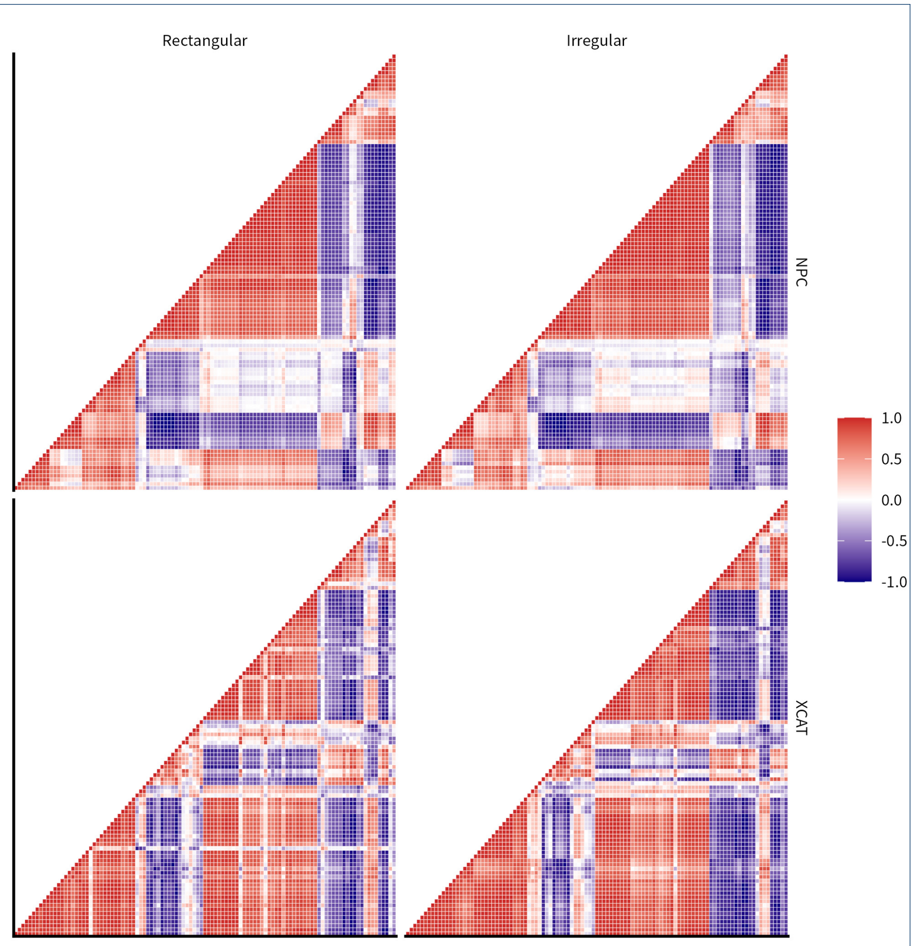

Figure 17 Heatmaps showing Spearman's correlation coefficients calculated between radiomic features as function of dataset and initial mask with AP segmentation. The feature order in correlation matrics was set according to the correlation coefficients for ConSeg with irregular mask in NPC dataset.

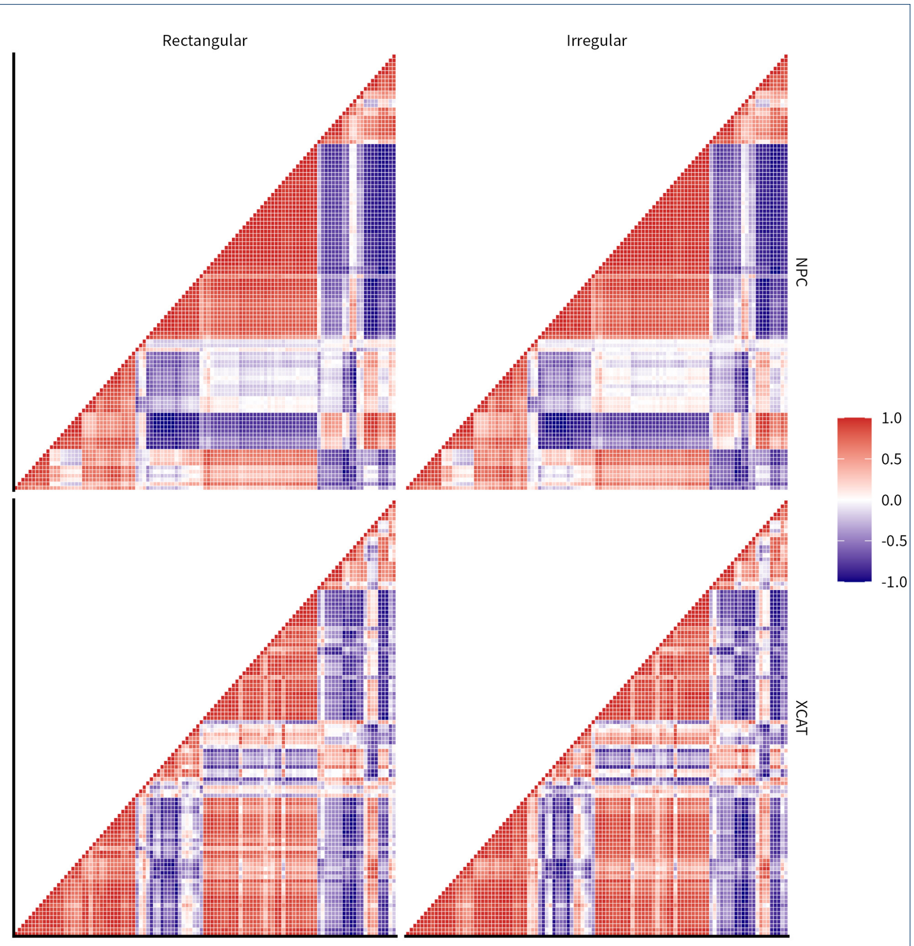

Figure 18 Heatmaps showing Spearman's correlation coefficients calculated between radiomic features as function of dataset and initial mask with ST segmentation. The feature order in correlation matrics was set according to the correlation coefficients for ConSeg with irregular mask in NPC dataset.

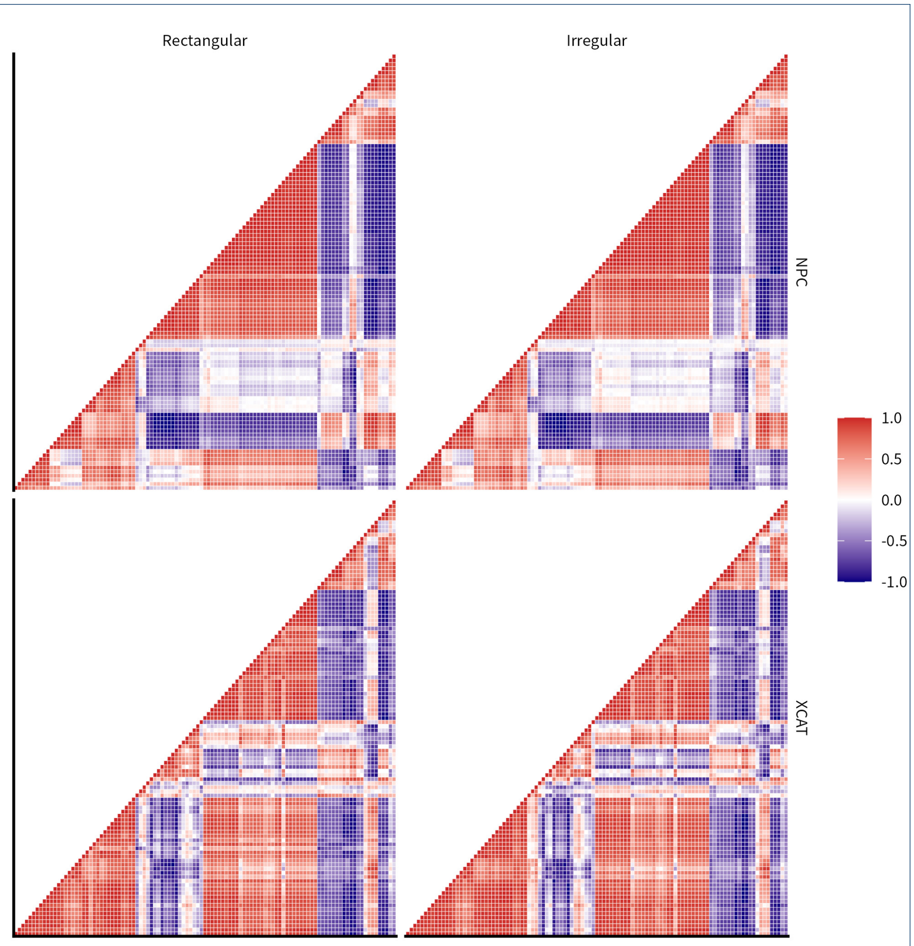

Figure 19 Heatmaps showing Spearman's correlation coefficients calculated between radiomic features as function of dataset and initial mask with 41MAX segmentation. The feature order in correlation matrics was set according to the correlation coefficients for ConSeg with irregular mask in NPC dataset.
